# The evolution of media reportage on GMOs in Ghana following approval of first GM crop

**DOI:** 10.1080/21645698.2024.2365481

**Published:** 2024-06-10

**Authors:** Joseph Opoku Gakpo, Dennis Baffour - Awuah

**Affiliations:** a Genetic Engineering and Society Center, North Carolina State University, Raleigh, North Carolina, USA; b Department of Agricultural and Human Sciences, North Carolina State University, Raleigh, North Carolina, USA; c Department of Nuclear Agriculture and Radiation Processing, University of Ghana, Accra, Ghana

**Keywords:** Food security, GM crops, GMOs, journalists, media coverage, science communication

## Abstract

Ghana’s parliament in 2011 passed the Biosafety Act to allow for the application of genetically modified organism (GMO) technology in the country’s agriculture. In a vibrant democracy, there have been extensive media discussions on whether GM crops will benefit or harm citizens. In June 2022, the state GMO regulator, the National Biosafety Authority (NBA), approved the country’s first GM crop (Bt cowpea) for environmental release, declaring the crop does not present an altered environmental risk or a food/feed safety concern. This study identified 3 of the country’s most vibrant digital news outlets and did a content analysis of all GMO stories reported 18 months pre- and post-approval to assess whether the approval changed the focus of GMO issues the media reports on. 91 articles were identified. The results show media reports on the likely impact of GMOs on the country’s food security shot up after the approval. However, media reports on the possible health, sociocultural, and environmental impact of GMOs declined. We observe the media and the public appear interested in deliberations on how the technology could address or worsen food insecurity and urge agricultural biotechnology actors in Ghana to focus on that in their sensitization activities.

## Introduction

The media’s influence in shaping consumer decisions and policies is far-reaching, impacting individuals, businesses, governments, and society as a whole. By framing issues, disseminating information, shaping public opinion, and advocating for change, the media plays a central role in shaping the cultural, social, economic, and political landscape of societies.^1–3^ Recognizing and understanding the media’s influence is essential for consumers, policymakers, and stakeholders seeking to navigate an increasingly media-saturated world. The media help drive consumer preference and influence their purchasing decisions in all sectors, including food systems. The content the media puts out to the public hugely determines consumers’ decisions on food in terms of cost, perceived quality, and appearance.^4^ They collect and disseminate information while providing oversight of regulatory institutions’ operations and are usually eager to influence opinions on public issues.^3,5^ Indeed, most government intervention programs, including those in agriculture, gained prominence when they were adequately publicized by the media.^2^ Across the African continent, agricultural reportage is featured in magazines, newspapers, social media, online news portals, and other media outlets, and although agricultural reporting is not as popular as sports, politics, and crime, it can increase awareness of agriculture and national development.^6^ Agricultural reportage in Africa is however usually centered on fertilizer use, crop improvement, markets, climate, irrigation, post-harvest and storage processes, transport facilities, agricultural machinery, and credit and loans,^7^ with few contents on agricultural biotechnology.

Media coverage of agricultural, health, environmental, and related science and technology issues influences people’s decisions on the foods they consume.^1,8,9^ The media’s coverage of the application of genetically modified organism (GMO) technology in food production is having a similar impact as the media’s portrayal of the technology is significantly influencing consumer perceptions, attitudes, and behaviors toward it.^10^ The way the media reports on GM crops, along with individuals’ general attitudes toward the media, appears to influence how people perceive the risks associated with GMOs in their food.^11,12^ The media plays a significant role in shaping public perceptions about GMOs because the technology is a relatively new and intricate concept, characterized by a cloud of uncertainty that enhances its news value.^11^ The media serves as a primary communication channel for disseminating information about GMOs as the public turns to it for clarity on the complex scientific details of the technology that may not be easily comprehensible to the general public.^13^ This means a complex interplay between media representation, journalistic credibility, and public perception, is one key factor that determines the fate of GMOs in societies.^10,14^

Lukanda et al.^15^ identifies journalists as key actors and interested parties in the controversial GMO debates, equating their role in the sector to that of scientists, multinational companies, and non-governmental organizations. The media functions at the intersection of researchers, politicians, and business interests, exerting a considerable influence on societal perceptions regarding genetic engineering, its value, and its application in society. It plays a crucial role in conveying the current state of knowledge regarding science and technological innovations like GMOs to the public, delineating both the risks and benefits while debunking misinformation and abstract facts about the technology.^16^

The impact media coverage of GMOs has on public attitude toward the technology goes a long way to help shape government policy, as public opinion can lead to increased pressure on policymakers to take a strong stance for or against imposing restrictions.^14,15^ Media coverage can influence the political agenda, as policymakers usually seek to respond to media narratives and frame their policies in ways that resonate with the public. In 2011, stakeholders in the agricultural sector in Zambia who engaged in media discussions on GMOs failed to engage in a meaningful dialogue that would have allowed for a holistic analysis of the technology, resulting in the decision of the government to reject GM corn aid from the USA at the height of a famine that was killing many citizens.^8^ The Zambian media’s challenge in effectively communicating information about GMOs in the region, the author says, was because the communication often took the form of a “debate” rather than adopting the more conventional journalistic modes of scientific knowledge dissemination and “civic education.”

Similar examples of the media influencing governments to develop negative attitudes toward GMOs have been reported in Europe and Asia. In 2016, Russia’s upper legislative chamber approved a law that established “a ban on the cultivation and breeding of genetically modified plants and animals on the territory of the Russian Federation, with the exception of their use for examinations and scientific research” (Council of the Federation,^17^ p.1). The law, which empowered the government to strengthen measures aimed at monitoring GMO-related activities in the country and preventing the release of GMOs into the environment, also received expressions of support and approval from the legislative assemblies of eight Russian provinces.^18^ The framing of media content was closely linked to a noticeable heightening in negative public sentiments about GMOs in Russia in the years preceding the ban imposed in 2016^14^ and this helped build public support for it. Framing of media content through the negative toning of stories and prominence given to specific perspectives about the technology contributed actively to the surge in negative public opinions about GMOs.^14^

According to Sohi et al.,^9^ 26 countries worldwide, including 19 in the European Union, currently have partial or full bans on GMOs, with one of the top reasons for this move being heavy public perception about possible negative health effects of the technology. A study in the UK and Spain by Vilella-Vila and Costa-Font^12^ revealed media coverage of GM foods has mainly centered on the risks and potential hazards to public health, and that, coupled with individual attitudes toward journalism, are interconnected with people’s opinions of the technology. Orchestrated campaigns organized by opponents of GMOs in Europe and Africa have encouraged negative media coverage and negative public attitudes toward the technology, and “there is still a substantial amount of opposition that may be driven or exacerbated by media-originated misinformation”^19^ p. 2). Yang et. al.’s^20^ analysis of Chinese media coverage of genetically modified vitamin A-rich Golden Rice found that although only one-third of the articles evoked negative tendencies toward GM crops, the stories had been written using the kind of wording that sparked fears about GMOs. Despite the nearby Philippines approving Golden Rice in 2021,^21^ China is still yet to approve the variety.

The key role the media plays in influencing consumer attitude and public policy about food, and GMOs in particular, makes it imperative for those involved in the agricultural biotechnology sector to pay attention to what the media reports on the technology. This study will examine how the media in Ghana, a country where efforts are ongoing to commercialize GMOs, is covering the technology.

## GMOs in Ghana

Ghana’s parliament approved the Biosafety Act (Act 831) in 2011 to permit the testing, production, and commercialization of GM crops in the country.^22,23^ The law mandates the establishment of the National Biosafety Authority (NBA) to ensure an integrated approach to regulating GMOs and other modern biotechnology products and ensure adequate protection and safety.^23^ Since then, a number of trials have been ongoing to develop GM crops locally. The Council for Scientific and Industrial Research (CSIR) has submitted applications to the NBA for approvals to conduct trials of various GM crops including the Nutrient Enhanced Sweet Potato; Nitrogen-use Efficient, Water-use Efficient, Salt Tolerant (NEWEST) rice; Podborer Resistant Cowpea (Bt cowpea); and Bollworm resistant cotton (Bt cotton).^24^ All these works were discontinued due to lack of funding, except that on GM cowpea.

Cowpea is a high-protein orphan crop consumed by an estimated 200 million people in Africa daily, making it an important crop to the continent’s food security.^25^ It’s usually cooked and eaten with carbohydrate sources like plantain and rice and is usually referred to as the poor man’s meat. Cowpea farmers in Ghana often apply pesticides up to eight times during the 12-week life cycle of the crop due to significant infestations by the Maruca pod borer pest.^26^ This practice leads to pollution of the atmosphere, poses risks of poisoning to farm workers, and ultimately reduces farmer profits. The genetically modified cowpea, also known as the Pod Borer Resistant cowpea or Bt cowpea, was developed by introducing a gene from the naturally occurring bacteria *Bacillus thuringiensis* (Bt), and this modification grants the cowpea inherent immunity to the Maruca pod borer pest, reducing the need for pesticide application to just two sprays.^27^ Work on GM cowpeas started in Ghana in 2015 and the National Biosafety Authority eventually approved it for environmental release in 2022.^22,28^ The GM cowpea development was the result of collaborative works between Australian, American, and Ghanaian scientists, with funding from USAID, Bayer, and other international institutions through the Kenyan-based African Agricultural Technology Foundation.^29^). Ghanaian scientists at the Savanna Agricultural Research Institute (SARI) of the Council for Scientific and Industrial Research in Nyankpala, Tamale, led the processes. A 2018 study forecasted that the GM crop will grow the nation’s cowpea sector by nearly 10% annually over the next six years and add US$52 million to the cowpea production economy by 2025 if it was commercialized as planned in 2019.^30^ Ghana is the second country in the world to approve GM cowpea after Nigeria approved the GMO in 2019.^31^

This study analyzes media publications about GMOs one-and-half years before the approval (January 2021 to June 2022), and one-and-half years after the approval (July 2022 to December 2023). We assess whether the approval changed the focus of GMO issues the media reports on. Although the approval was specifically for GM cowpea, it appears the move sparked broad conversations about GMOs in the media since it was the first approved GMO in the country. The study will offer insight into GMO issues that the media finds exciting. Ghanaians rely heavily on the media for information on GMOs, with 57.5% of respondents to a survey saying print media and online articles are their preferred sources for the acquisition of information on GMOs.^32^ Understanding how the media covers the technology will be crucial if GMO actors can appropriately sensitize the public on the technology.

## Methodology

This mixed method study purposively identified three of the most vibrant digital news outlets in Ghana and did a content analysis of all GMO stories reported over three years from January 2021 to December 2023. We sourced data for this study from Ghanaweb.com, the most-read news website in Ghana; Myjoyonline.com, the second most-read news website in Ghana, and Graphic.com.gh, the digital platform of the most influential newspaper in Ghana, Daily Graphic^33–35,^^36^ We used 8 keywords to search for all GMO-focused stories reported on these news websites over the period; GMO, GMOs, GM foods, GM Crops, Biotech crops, Biotechnology crops, Genetically modified crops, and Genetically Engineered Crops. We read through each of the articles and coded them manually, as was done by Lynas et al.,^19^ into one of 5 dominant issue categories; human health articles, food security articles, environment articles, sociocultural articles, and economics articles. The majority of the articles had elements from more than one of the 5 focus areas. But the dominant one that made up the majority of the content was what we used in categorizing the articles, just as Lukanda et al.^15^ did in undertaking a similar media coverage of GMOs study.

Lynas et al.^19^ describe human health GMO articles as those that focus on how GM crops relate to human health, including claims and counter-claims about their impact on consumers’ cancer and obesity statuses, as well as possible positive impacts on health in areas of biofortification and reduction in aflatoxins. Busuulwa et al.^37^ describe economics-related GM articles as those with keywords like trade and price, and we broadened that definition to include articles that discuss how GMOs may improve or negatively impact the wealth and economic power of individuals and countries. Environment GMO articles are those that discuss how the cultivation of GM crops impacts cross-pollination, soils, wildlife, and the environment generally.^19^ Availability, access, utilization, and stability are the four dimensions of food security, and food security exists when people have sufficient access to safe and nutritious meals at all times.^38,39^ We thus defined food security GMO articles as those that discuss crop yield and productivity, biotic and abiotic stress tolerance of varieties, and crop nutrition, all of which positively or negatively impact the availability and accessibility of food. Sociocultural contexts describe how society, culture, and systems impact human life.^40^ Based on this, we describe sociocultural articles as those that elaborate on the possible impacts of GMOs on society and culture outside the above-mentioned four areas. Articles linking GMOs to cultural norms and beliefs, religious practices, habits, lifestyles, and related areas were placed in this category. We present the results below in volumes and percentages for each of the categories.

## Results

**Table 1.** Total number of publications.

Media House	No of Publications Pre-approval	No of Publications Post-approval	Total
Myjoyonline	19	18	37
Ghanaweb	14	21	35
Graphic	4	15	19
**Total**	**37**	**54**	**91**

**Figure 1.**
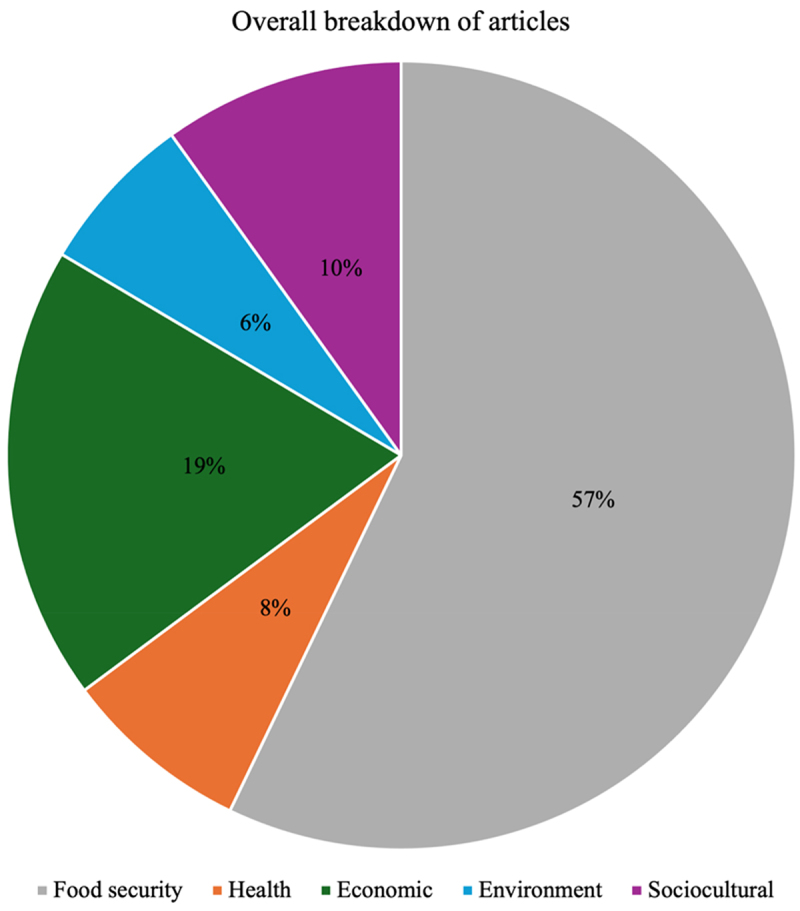
Breakdown of all articles into various issues.

The three media houses published 91 GMO-focused articles between January 2021 and December 2023, as shown in Table 1. Myjoyonline recorded the highest number of published GMO articles over the three years, recording 40.66% of all the articles published, with Ghanaweb coming second with 38.46%, whilst Graphic had 20.88%. Also, overall, 57.14% of all articles published by the three media houses were focused on food security, 7.69% focused on health, 18.68% focused on economics, 6.59% focused on environment, and 9.89% focused on sociocultural issues, as shown in Figure 1. Whilst Myjoyonline published fewer articles post-approval compared to pre-approval at a rate of 48.65% and 51.35%, respectively, Graphic and Ghanaweb recorded the opposite. 60% of all Ghanaweb GMO articles were published post-approval compared with 40% pre-approval, and Graphic’s ratio was 78.95% post-approval and 21.05% pre-approval.

62.43% of all Myjoyonline articles focused on food security, 2.63% focused on health, 15.94% focused on economics, 8.19% focused on the environment, and 10.82% focused on sociocultural issues. 52.38% of all Ghanaweb articles focused on food security, 13.20% focused on health, 13.20% focused on economic issues, 10.72% focused on environmental issues, and 10.72% focused on sociocultural issues. 48.34% of all Graphic GMO stories focused on food security issues, 6.67% focused on health issues, 29.17% focused on economic issues, 0% focused on environmental issues, and 15.84% focused on sociocultural issues.

**Figure 2.**
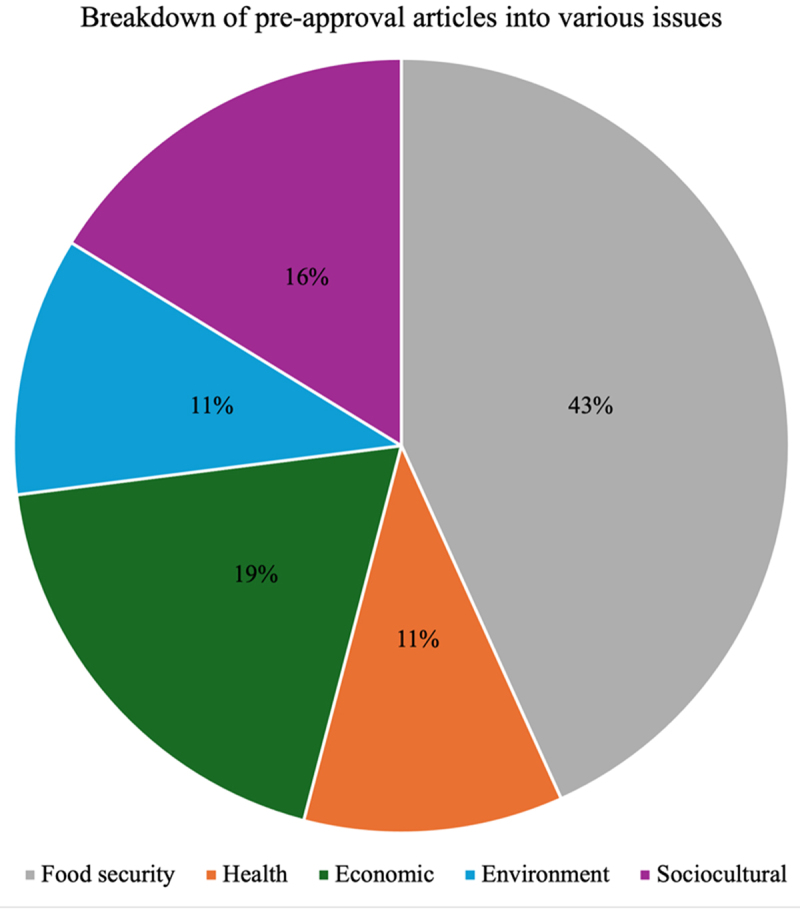
Breakdown of all pre-approval articles into various issues.

**Figure 3.**
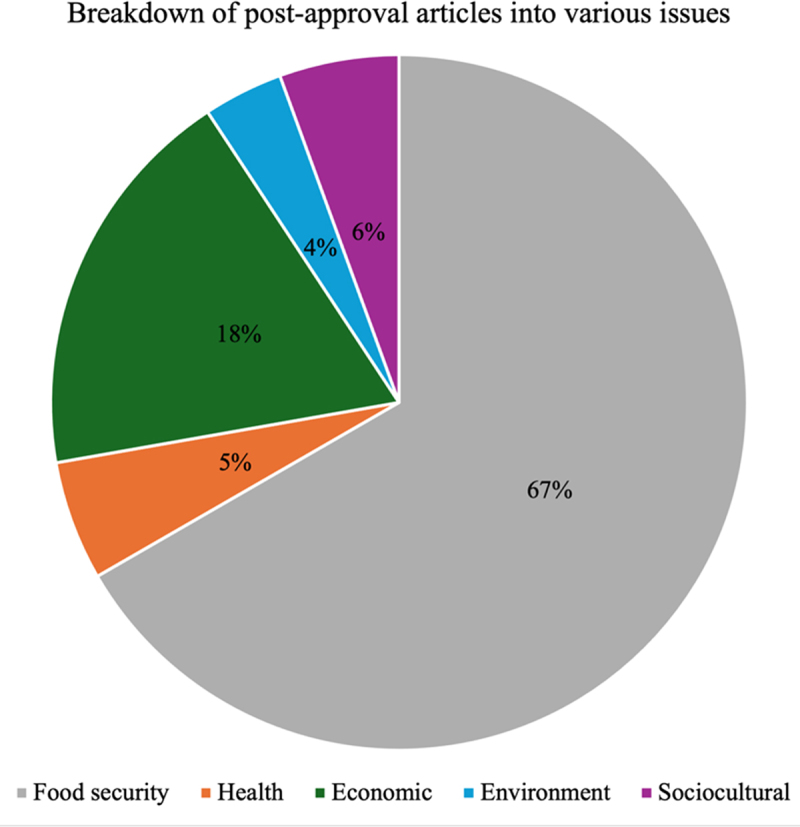
Breakdown of all post-approval articles into various issues.

As shown in Figure 2, 43.24% of all GMO articles published by the three media organizations before the NBA approval focused on the theme of food security. That figure shot up to 66.67% in the period post-approval, as shown in Figure 3. The volume of health-focused articles prior to approval was 10.81%, and that figure fell to 5.55% post-approval. All the health-focused articles raised concerns about the possible negative impact of GMOs. The volume of economic-focused articles remained almost the same despite a slight drop post-approval, with the pre and post-approval percentages being 18.92% and 18.52%, respectively. The volume of environment-focused articles dropped from 10.81% to 3.70%, whilst the volume of sociocultural articles dropped from 16.22% to 5.56%.

Whilst 52.63% of all Myjoyonline articles published pre-approval focused on food security, the figure post-approval was 72.22%. Health-focused articles declined from 5.26% to 0%, economic-focused articles decreased from 26.32% to 5.56%, environment-focused articles increased from 5.26% to 11.11%, and sociocultural articles increased slightly from 10.53% to 11.11%. Ghanaweb saw an increase in food security articles from 28.57% to 76.19% post-approval, whilst health-focused articles decreased from 21.43% to 4.76%, economic articles increased from 7.14% to 19.05%, environment articles from 21.43% down to 0%, and sociocultural articles from 21.43% to 0%. Graphic is the only platform of the three to have recorded a decrease in reportage on food security post approval from 50% to 46.67%, although that was a slight decrease. Health articles in Graphic increased from 0% to 13.33%, economic articles increased from 25% to 33.33%, environmental articles remained the same at 0%, and sociocultural articles declined from 25% to 6.67%.

## Discussions

Majority of all stories published by the three media houses (57%) were focused on food security, with the four other issues sharing the rest of the news space. This agrees with a similar study conducted in another African country, Uganda, by Lukanda et al.,^15^ which analyzed GMO-focused stories reported in two national dailies over four years. The study revealed the majority of all articles (44%) focused on food security, whilst stories about regulation, health risks, environmental effects, GMO labeling, and other issues, constituted the rest. The huge media focus on food security issues in reporting on GMOs was expected because Africa, including Ghana, remains one of the most food-insecure places in the world. Whilst 20.2% of the population in Africa faces hunger, only 9.1% of the population in Asia does, 8.6% in Latin America and the Caribbean, 5.8% in Oceania, and less than 2.5% in Northern America and Europe.^41^ Climate change, aftershocks of the COVID-19 pandemic, and conflicts in and outside of the continent have exacerbated food insecurity challenges on the continent, resulting in increasing unavailability of food to a good number of people, with at least one in every five Africans going to bed hungry.^42–44^ Food prices in Ghana have continued to soar since the COVID-19 pandemic, and an estimated 39% of the population faced moderate or severe food insecurity in 2022.^45–47^

As De Maeyer,^48^ observes, there is a higher likelihood that journalists and editors will write a story and publish a story respectively if the story impacts a large number of people or if the content has a high national interest. Food insecurity is an issue that affects a large number of people in Ghana so journalists are more motivated to frame stories and set agendas in that direction if they can, and they appear to have done a lot of that in their work on GMO articles. Without listeners, readers, and viewers, it is impossible to practice journalism and so audience interest is another key driver that shapes the stories the media reports on.^49^ A good number of Ghanaians are food insecure and are likely to be interested in the role technologies like GMOs could play in improving or worsening food security, hence the huge journalists’ interest in such stories. Other studies have also backed the huge media interest in food security issues whilst reporting on GMOs in Africa. Outram,^16^ after interviewing journalists and academics from across Africa reported that “food-security issues were felt to be highly relevant to the discussion of genetic science and biotechnology within Africa” (p.12). Framing refers to a communication practice where journalists help the public construct meanings of issues by determining which perspectives take precedence and are most frequently highlighted in their coverage.^1^ Whether through headlines, articles, or broadcasted narratives, the deliberate selection of words by news outlets can evoke specific emotions, highlight particular aspects of a story, and even shape the overall narrative direction, which then shapes audience opinions, attitudes, and perception of the reported information.^50,51^ On the subject of GMOs, the Ghanaian media frames the subject as a food security issue.

Out of the three platforms, state-owned Graphic published the least number of food security articles, with just 48.34% of their articles being food security-focused, whilst Myjoyonline and Ghanaweb, which are both private media houses, had 62.43% and 52.38% respectively. Although Graphic.com.gh and its mother company Daily Graphic generate enough revenue to run without having to rely on direct government funding, it receives a considerable share of state advertising from government ministries and agencies to compensate for the absence of direct government subsidies.^34^ That is how it survives. It thus does not have to pay that much attention to the interest of target audiences in the kind of stories it produces since advertising revenue will usually come from the government. That is probably why its reportage on GMOs doesn’t appear to reflect where the public interest lies, which is food security.

8.18% of all the articles were health-focused, and 7.26% focused on the environment. These figures are similar to those reported by Lukanda et. al.^15^ in a Ugandan study, which showed 13% and 9% of articles were health and environment-focused, respectively. Both countries appear to be exhibiting similar attributes in how journalists cover GMOs although they are at different stages in the efforts to incorporate the technology in their food systems. Ghana approved a law in 2011 to legalize the growing of GMOs, but Uganda has yet to approve such legislation (Lukanda et. al.^15^; USDA^52^) Ghana has given environmental release approval for at least one GM crop, whilst Uganda has yet to approve any such crop (Lukanda et. al.^15^; USDA,^15^).

18.2% of all the articles analyzed in this study were economics-focused. This is contrary to the results of a related study by Busuulwa et. al.^37^ that analyzed keywords in GMO-focused articles published in 6 African countries between 2016 and 2019. That study revealed economic themes were most dominant. The contradiction could be because both studies analyzed media reports from two different periods, with ours focusing on 2021 to 2023, while that study focused on 2016 to 2019.

Our study also found that media articles on the more controversial aspects of GM crops (health and environmental issues), declined following the approval given by the National Biosafety Authority (NBA) for the first GMO. Media reports on health issues relating to GM technology declined from 10.81% to 5.55%, and environmental issues from 10.81% to 3.70% following the approval, a five and seven percent gap reduction, respectively. The approval document released by the NBA addressed both subjects. The authority said its analysis of agronomic data from multiple years of testing the GM cowpea variety revealed that it did not exhibit unintended or unexpected effects on plant growth habit, general morphology, vegetative vigor, or grain yield, and there were no indications that the GM crop would have a higher impact on biodiversity, compared to conventional cowpea varieties.^53^ The document also said there are no safety concerns regarding the development process as no new hazards have been identified with the gene transfer process. It said the GM cowpea variety is unlikely to be toxic or allergenic to mammals, and the gene introduced from bacteria would not cause antibiotic resistance in humans, and will not result in altered impacts on non-target organisms, including humans.

We are unable to conclude whether journalists’ reduced interest in the controversial health and environment GMO issues was the result of the NBA’s pronouncements in the approval document that the technology will have no new negative health and environmental implications. As McFadden and Lusk^54^ observe, many people won’t change their minds about genetically modified foods and global warming when they get new information on the technology and some even develop more entrenched positions that GMOs are unsafe after reading scientific information confirming their safety. Meanwhile, other studies by Foundation for Future Agriculture Research et. al,^55^ Sleboda and Lagerkvist,^56^ Pham and Mandel,^57^ Kato-Nitta et al.,^58^ and Stanton et al.^59^ showed exposure to tailored information on GMOs and genome editing, can cause a shift toward more favorable perceptions, although at varying rates. After being compelled to read a reliable scientific statement regarding the safety of GMOs from organizations like the World Health Organization, Royal Society of Medicine, American Academy of Arts and Sciences, National Academy of Sciences, and American Medical Association, consumers experienced a notable reduction in their apprehensions regarding its potential negative health impacts or cancer-causing properties.^59^ More in-depth studies are needed to establish a clearer understanding of the impact the NBA’s pronouncements had on journalists.

While the total number of stories published pre-approval was 37, the number of stories published post-approval was 54. That represents a 45% increase in the number of stories across the three media platforms. Stories about GMOs continue to gain traction on mainstream media platforms across the world, as media interest in the subject increases. GMO stories on media platforms globally increased from 20,300 in 2018 to 34,000 in 2019 and further to 48,600 in 2020, with the extent of the reach of these stories increasing from 1.8 billion to 3.7 billion.^60^ It appears Ghana is following the worldwide trend with increasing media interest in science issues like GMOs.

## Conclusions

Despite assurances from government regulatory institutions and scientific bodies that agricultural biotechnologies like GMOs and CRISPR gene editing do not cause new safety concerns compared to their conventional counterparts, consumers often remain skeptical.^61,62^ Such skepticism is driven not by ignorance but by perceived risks associated with technologically advanced food production methods, and the media plays a key role in encouraging such perception.^10,19^ In Russia, China, Europe, and other parts of Africa, the media’s continued focus on the perceived risks associated with GM crops has been identified as one of the key developments that encouraged the public to develop negative sentiments against the technology.^14,19^ The media’s key role in influencing consumer attitudes and public policy about food makes it imperative for scientists, industries, government institutions, and non-governmental organizations working on GMOs to actively engage the media on the technology. By working closely with the media, technology promoters can ensure that accurate and informative content is disseminated, helping to dispel any misconceptions or misinformation about GMOs that may exist. We note the Ghanaian media and the public appear interested in deliberations on how the technology could address or worsen food security in the country and urge GMO actors to increase media engagement on such topics.

The National Biosafety Authority must also increase media engagement on biosafety issues. The Biosafety Act 2011 says, “The Authority shall promote public awareness participation and education concerning biosafety matters for the benefit of the people of the Republic through … public lectures, seminars, and workshops”^23^ p. 19). The NBA should prioritize the use of the media in its educational activities. Engaging with the media can help amplify the NBA’s message to reach a wider audience, increasing awareness and understanding of the issues at hand. The Biosafety Authority also needs to do more to communicate the vigorous regulatory processes that GMOs go through, as well as the basics of risk assessment to increase public confidence in the regulatory process. In seeking to ensure fair reporting, journalists would usually try to balance their reports by giving equal weight to comments by scientists who speak in favor of the technology and campaigners kicking against it. The NBA can serve as an arbiter providing third-party judgments on the technology in the court of public opinion.

As Vigani^63^ observes, the mass media serve as intermediaries between citizens and governments during the formulation of national regulations concerning GMOs and are utilized by various interest groups aiming to sway consumer attitudes and influence policy outcomes. The mass media play a vital role in mediating the exchange of ideas, opinions, and information between policymakers, stakeholders, and the general public. By providing a platform for debate, analysis, and discussion, media outlets enable citizens to engage with complex issues surrounding GMO regulation, fostering a more informed and participatory democratic process. Furthermore, the mass media serve as battlegrounds where various interest groups, including agricultural organizations, environmental activists, scientific communities, and industry stakeholders, vie for attention and seek to shape public discourse and policy outcomes regarding GMOs. The media serves as a powerful intermediary between technical information on GMOs and the public, and journalists exert significant influence on how issues are perceived and understood within society. Through the media, the NBA can build credibility and trust with the public, ultimately leading to greater public acceptance of the authority’s decisions on GMOs.

## Limitations of Study

The stories journalists tell can be biased by current events happening at any point in time. It is thus possible that the evolution of the focus of GMO issues that the media reports on may have been influenced by developments in Ghana at various points in time. The COVID-19 pandemic brought up repeated discussions about food insecurity and may have contributed to the heightened conversation about food insecurity issues, when the media reported on GMOs between 2021 and 2023. We were unable to eliminate such biases from the data. Also, the study only did content analysis of media reports, and does not provide enough data to draw conclusions on how the approval of the first GMO changed the perception of journalists on the technology. We recommend future studies collect quantitative and qualitative data from Ghanaian journalists on their perception of the GMO technology and how the approval impacted the way they cover the technology. We also recommend similar in-depth studies of farmers’ and consumers’ perceptions be undertaken.
